# Many-core algorithms for high-dimensional gradients on phylogenetic trees

**DOI:** 10.1093/bioinformatics/btae030

**Published:** 2024-01-18

**Authors:** Karthik Gangavarapu, Xiang Ji, Guy Baele, Mathieu Fourment, Philippe Lemey, Frederick A Matsen, Marc A Suchard

**Affiliations:** Department of Biomathematics, David Geffen School of Medicine at UCLA, University of California, Los Angeles, Los Angeles, CA, United States; Department of Mathematics, School of Science & Engineering, Tulane University, New Orleans, LA, United States; Department of Microbiology, Immunology and Transplantation, Rega Institute, KU Leuven, Leuven, Belgium; Australian Institute for Microbiology and Infection, University of Technology Sydney, Ultimo, NSW, Australia; Department of Microbiology, Immunology and Transplantation, Rega Institute, KU Leuven, Leuven, Belgium; Public Health Sciences Division, Fred Hutchinson Cancer Research Center, Seattle, WA, United States; Department of Statistics, University of Washington, Seattle, WA, United States; Department of Genome Sciences, University of Washington, Seattle, WA, United States; Howard Hughes Medical Institute, Fred Hutchinson Cancer Research Center, Seattle, WA, United States; Department of Biomathematics, David Geffen School of Medicine at UCLA, University of California, Los Angeles, Los Angeles, CA, United States; Department of Biostatistics, Jonathan and Karin Fielding School of Public Health, University of California, Los Angeles, Los Angeles, CA, United States; Department of Human Genetics, David Geffen School of Medicine at UCLA, Los Angeles, CA, United States

## Abstract

**Motivation:**

Advancements in high-throughput genomic sequencing are delivering genomic pathogen data at an unprecedented rate, positioning statistical phylogenetics as a critical tool to monitor infectious diseases globally. This rapid growth spurs the need for efficient inference techniques, such as Hamiltonian Monte Carlo (HMC) in a Bayesian framework, to estimate parameters of these phylogenetic models where the dimensions of the parameters increase with the number of sequences *N*. HMC requires repeated calculation of the gradient of the data log-likelihood with respect to (wrt) all branch-length-specific (BLS) parameters that traditionally takes $O(N^{2})$ operations using the standard pruning algorithm. A recent study proposes an approach to calculate this gradient in O(N), enabling researchers to take advantage of gradient-based samplers such as HMC. The CPU implementation of this approach makes the calculation of the gradient computationally tractable for nucleotide-based models but falls short in performance for larger state-space size models, such as Markov-modulated and codon models. Here, we describe novel massively parallel algorithms to calculate the gradient of the log-likelihood wrt all BLS parameters that take advantage of graphics processing units (GPUs) and result in many fold higher speedups over previous CPU implementations.

**Results:**

We benchmark these GPU algorithms on three computing systems using three evolutionary inference examples exploring complete genomes from 997 dengue viruses, 62 carnivore mitochondria and 49 yeasts, and observe a >128-fold speedup over the CPU implementation for codon-based models and >8-fold speedup for nucleotide-based models. As a practical demonstration, we also estimate the timing of the first introduction of West Nile virus into the continental Unites States under a codon model with a relaxed molecular clock from 104 full viral genomes, an inference task previously intractable.

**Availability and implementation:**

We provide an implementation of our GPU algorithms in BEAGLE v4.0.0 (https://github.com/beagle-dev/beagle-lib), an open-source library for statistical phylogenetics that enables parallel calculations on multi-core CPUs and GPUs. We employ a BEAGLE-implementation using the Bayesian phylogenetics framework BEAST (https://github.com/beast-dev/beast-mcmc).

## 1 Introduction

Genomic sequencing has become a critical tool in monitoring the evolution and spread of infectious pathogens to inform public health interventions, as the unprecedented number of genomes sequenced to monitor the emergence and growth of variants during the ongoing SARS-CoV-2 pandemic demonstrates (Oude Munnink *et al.* 2021, Brito *et al.* 2022). This has created the need for statistical phylogenetic methods that can be used to derive useful insights in a timely manner from these large molecular sequence alignments. Within a Bayesian framework, such analyses typically employ Markov chain Monte Carlo (MCMC) methods such as the random walk Metropolis-Hastings (MH) algorithm (Metropolis *et al.* 1953, Hastings 1970) to simultaneously infer the discrete tree topology and continuous branch-length-specific (BLS) parameters such as the branch lengths (or correspondingly, node heights) and branch-specific evolutionary rates. However, the number of possible tree topologies increases super-exponentially, while the dimensionality of continuous BLS parameters further increases linearly with the number of sequences in the alignment. There exist state-of-the-art MCMC algorithms such as Hamiltonian Monte Carlo (HMC) that use Hamiltonian dynamics to traverse the parameter space more efficiently compared to a random walk proposal distribution (Neal 2011). Using HMC enables phylogenetic methods to more efficiently infer continuous high-dimensional BLS parameters, sparing valuable compute time to search the discrete space of possible tree topologies. Nonetheless, this efficiency comes with the cost of needing to calculate the gradient of the molecular sequence alignment log-likelihood function with respect to (wrt) all BLS parameters, an aspect that remains computationally intensive.

One generally assumes that the individual alignment sites arise conditionally independently from a continuous-time Markov chain (CTMC) process acting along the branches of an estimable evolutionary tree relating the *N* sequences, where a branch length and, often, an associated evolutionary rate-scalar characterize the branch-specific processes. Under this CTMC model, Felsenstein’s pruning algorithm (Felsenstein 1981) renders the calculation of the likelihood computationally tractable. The pruning algorithm calculates the probability of only the observed sequence data below each internal node in the tree in a single post-order traversal that visits each node in a descendant-to-parent fashion. By efficiently reusing these partial likelihood vectors from previously visited nodes, the algorithm reduces the computational complexity of calculating the likelihood to just O(N) with respect to the number of sequences *N*. The same algorithm can be used to calculate the gradient of the log-likelihood wrt a *single* BLS parameter by substituting the CTMC transition probability matrix along one branch with its derivative (Kishino *et al.* 1990, Bryant *et al.* 2005, Kenney and Gu 2012). In this manner, calculating the gradient of the log-likelihood wrt all BLS parameters requires $O(N^{2})$ operations. Several software packages, such as RaxML (Stamatakis 2014) and GARLI (Zwickl 2006), utilize this pruning algorithm approach for low-dimensional gradient calculations by selectively optimizing BLS parameters directly affected by a tree rearrangement. For instance, they may optimize BLS parameters for only the three local branches adjacent to the point of insertion following a topological change. Until recently, the computational cost of evaluating the gradient of the log-likelihood wrt *all* BLS parameters has proved too prohibitive for the use of high-dimensional gradient-based samplers or optimizers in statistical phylogenetics.

Guindon and Gascuel (2019) and Ji *et al.* (2020), however, propose algorithms to calculate this gradient in linear-time. The former work assumes time-reversibility in the CTMC process and reuses the post-order partial likelihood vectors, while the latter makes no restrictions on the process. To accomplish this, Ji *et al.* (2020) introduce a pre-order traversal of the tree that involves visiting each node in a parent-to-descendant fashion after the post-order traversal. Combining the post- and pre-order partial likelihoods vectors calculated at each node with their branch-specific process derivative yields the whole gradient. Calculation of the pre-order partial likelihood vectors and their contribution to the gradient is straight-forwardly parallelizable in a multi-core CPU setting by dividing sites in the alignment into conditionally independent partitions that define separate computational tasks. These calculations may offer further parallelization across different rate categories for models that incorporate among-site rate variation often using a discrete approximation to the continuous gamma distribution (Yang 1996). Despite these existing parallelization schemes on CPUs, the widespread availability of specialized hardware such as graphics processing units (GPUs) opens up the possibility of further speeding up these calculations by many-fold.

GPUs were originally designed for image rendering but have since become ubiquitous for scientific computing. The availability of application programming interfaces such as OpenCL (Stone *et al.* 2010) and CUDA (Cook 2012) expanded the use of GPUs for general purpose computing beyond graphics processing. Since then, GPUs have been utilized to effectively parallelize computation for many statistical problems. Beam *et al.* (2016) leveraged GPUs to evaluate the gradient of the log-likelihood of a multinomial model, achieving nearly a 100-fold acceleration in inference via HMC. Similarly, Yang *et al.* (2023) employed GPUs in survival analyses with the Cox and Fine-Gray models, resulting in a 35–70-fold speed increase. Other problems benefiting from GPU acceleration include mixture modeling (Suchard *et al.* 2010), nonnegative matrix factorization (Zhou *et al.* 2010), and Bayesian multidimensional scaling (Holbrook *et al.* 2021). Additionally, GPU-optimized libraries have emerged in machine learning, such as XGBoost (Mitchell and Frank 2017) for gradient boosting and cuDNN (Chetlur *et al.* 2014) for deep learning.

Modern GPUs come equipped with thousands of simple cores, enabling them to carry out a vast number of lock-stepped calculations in parallel. In contrast, CPUs contain significantly fewer cores, yet each core can perform complex, independent tasks. This difference allows GPUs to deliver much higher parallelization compared to CPUs for fine-scale numerical operations on large blocks of data. Please refer to Supplementary Section S1.1 for a detailed description of the aspects of GPU architecture such as streaming multiprocessors (SM), global memory, shared memory, and ‘coalesced’ global memory transactions, which inform the design of our algorithms. In the phylogenetic setting, Suchard and Rambaut (2009) first described how to calculate the post-order partial likelihood vectors with fine-scale parallelization on GPUs and Ayres *et al.* (2019) report recent performance gains.

Building upon this prior work, we present here two novel algorithms that utilize GPUs to calculate the pre-order partial likelihood vectors and the gradient of the log-likelihood wrt all BLS parameters. We then benchmark computational performance in inferring BLS parameters using our algorithms on the GPU compared to the existing CPU implementation. We apply our algorithms to date the timing of the first introduction of West Nile virus into the continental United States under a codon model with branch-specific evolutionary rates, an inference task that was previously intractable. Finally, we highlight the limitations of our current approach and discuss future work to address these limitations and further exploit many-core algorithms for statistical phylogenetics.

## 2 Materials and methods

The molecular sequence alignment $Y=\left(Y_{1},\ldots ,Y_{C}\right)$ comprises *C* aligned columns or sites, where column data $Y_{c}=\left(Y_{1c},\ldots ,Y_{Nc}\right)\prime$ contains one homologous sequence character for each column c=1,…,C and each of the *N* taxa. Following standard practice since Felsenstein (1981), we assume that $Y_{c}$ are conditionally independent and identically distributed. Thus, it suffices to compute the post- and pre-order partial likelihood vectors and gradient using only the unique $Y_{c}$. We can appropriately reweigh these values based on the number of occurrences of a unique $Y_{c}$. Each aligned character *Y_nc_* for n=1,…,N exists in one of *S* possible states that we arbitrarily label {1,…,S}. For nucleotide alignments, the state-space size *S *=* *4; likewise amino acid and codon alignments yield *S *=* *20 and *S *=* *61, respectively. Additionally, for phylogeographic inference, *S* can be large, often comparable to codon models (Dudas *et al.* 2017, Lemey *et al.* 2020) and has eclipsed 200 in Markov-modulated CTMC models (Baele *et al.* 2021). We observe these characters from *N* taxa related by an (often) unknown phylogeny F. This phylogeny is a directed, bifurcating graph with *N* tip nodes corresponding to the taxa that we label (1,…,N), N−2 internal nodes that we label (N+1,…,2N−2) and one root node that we label 2N−1. Connecting each parent node to its child in F are 2N−2 edges or branches with branch lengths $\left(b_{1},\ldots ,b_{2N-2}\right)$ that we index via their child node number. Each branch length *b_i_* for i=1,…,2N−2 can measure the expected number of character substitutions along that branch or be the difference between the parent and child node heights measured in time-units multiplied by a (possibly branch-specific) evolutionary rate scalar. Without loss of generality, unrooted phylogenies are nested within this formulation by setting one of the branch lengths emerging from the root to zero.

An *S *×* S* infinitesimal generator matrix **Q** characterizes the CTMC process. Into this formulation, we further incorporate site-specific rate variation using the popular discretized models (Yang 1994) that modulate the CTMC for each column independently through a finite mixture of rate categories r={1,…,R} where each category corresponds to an overall rate scale *γ_r_* drawn with probability $P\left(\gamma_{r}\right)$. Thus under rate category *r*, the CTMC posits that substitutions at the parent and child nodes of any branch *i* in F are governed by an *S *×* S* finite-time transition probability matrix $P^{\left(r\right)}\left(b_{i}\right)=\left\{P_{st}^{\left(r\right)}\left(b_{i}\right)\right\}$, where

(1) $$P^{\left(r\right)}\left(b_{i}\right)=\text{exp}\;\left(\gamma_{r}b_{i}Q\right),$$

such that the $st^{\text{th}}$ element $P_{st}^{\left(r\right)}\left(b_{i}\right)$ is the probability of observed or unobserved state *t* at the child node of branch *i* given observed or unobserved state *s* at the parent node.

To form the partial likelihood vectors calculated during the post- and then pre-order traversals and understand how they relate to the sequence likelihood and then its gradient, we require some data augmentation. For column *c*, let *Y_ic_* for i=N+1,…,2N−1 represent the unobserved (latent) character states at the internal and root nodes. Further, we can divide the observed characters $Y_{c}$ at the tips into two disjoint sets wrt any node in F. Let $Y_{\left\lfloor ic\right\rfloor }$ be the observed characters at the tips descendant of node *i*, noting that $Y_{\left\lfloor 2N-1,c\right\rfloor }=Y_{c}$, and let $Y_{\left\lceil ic\right\rceil }=Y_{c}/Y_{\left\lfloor ic\right\rfloor }$ denote the observed characters at the tips not descendant from node *i*.

The post-order partial likelihood vector is denoted by $p_{\mathrm{irc}}=(p_{\mathrm{irc}1},\ldots ,p_{\mathrm{ircS}})\prime$, where $p_{\mathrm{ircs}}=P\left(Y_{\left\lfloor ic\right\rfloor }|Y_{ic}=s,\gamma_{r}\right)$ at node *i* for column *c* under rate category *r* with a realized rate scalar *γ_r_*. We compute these vectors using Felsenstein’s pruning algorithm via recursive application of

(2) $$p_{\mathrm{krc}}=\left[P^{\left(r\right)}\left(b_{i}\right)\right]p_{\mathrm{irc}}\;○\;\left[P^{\left(r\right)}\left(b_{j}\right)\right]p_{\mathrm{jrc}},$$

where node *k* is parent to node *i* and its sibling node *j* and  ○  signifies component-wise multiplication. If we let $\pi =\left(P\left(Y_{2N-1,c}=1\right),\ldots ,P\left(Y_{2N-1,c}=S\right)\right)\prime$ denote an arbitrary prior state distribution vector at the root that is often set equal to the stationary distribution of **Q**, then

(3) $$\begin{array}{c} P\left(Y\right)=\prod_{c=1}^{C}\sum_{r=1}^{R}P\left(Y_{c}|\gamma_{r}\right)P\left(\gamma_{r}\right)\\ =\prod_{c=1}^{C}\sum_{r=1}^{R}\left[p_{2N-1,rc}^{\prime }\pi \right]P\left(\gamma_{r}\right) \end{array}$$

yields the sequence likelihood. Suchard and Rambaut (2009) develop massively parallel algorithms for calculating matrices $P^{\left(r\right)}\left(b_{i}\right)$ for all *i* and *r* simultaneously and vectors $p_{\mathrm{irc}}$ for all *r* and *c* simultaneously on GPUs. Supplementary Figure S2 depicts the parallel thread-block design for the algorithm to calculate the post-order partial likelihood vectors described in Suchard and Rambaut (2009).

Ji *et al.* (2020) introduce the pre-order partial likelihood vector $q_{\mathrm{irc}}=\left(q_{\mathrm{irc}1},\ldots ,q_{\mathrm{ircS}}\right)\prime$, where $q_{\mathrm{ircs}}=P\left(Y_{ic}=s,Y_{\left\lceil ic\right\rceil }|\gamma_{r}\right)$ at node *i* for column *c* under rate category *r* and demonstrate how to compute these vectors recursively given the post-order partial likelihood vectors and transition matrices. Starting from the root and assigning $q_{2N-1,rc}=\pi$, we continue toward the tips via

(4) $$q_{\mathrm{irc}}=\left[P^{\left(r\right)}\left(b_{i}\right)\right]\prime \left\{q_{\mathrm{krc}}\;○\;\left[P^{\left(r\right)}\left(b_{j}\right)\right]p_{\mathrm{jrc}}\right\}.$$

The value of these pre-order partial likelihood vectors shines in the realization that

(5) $$P\left(Y\right)=\prod_{c=1}^{C}\sum_{r=1}^{R}\left[p_{\mathrm{irc}}^{\prime }q_{\mathrm{irc}}\right]P\left(\gamma_{r}\right)\;\text{for}\;\mathrm{any}\;\text{node}\;i,$$

and this insight enables a linear-in-*N*-time approach to evaluate the gradient of the log-likelihood wrt all BLS parameters

(6) $$\nabla \;\text{log}\;P\left(Y\right)=\left(\frac{\partial }{\partial b_{1}}\text{log}\;P\left(Y\right),\ldots ,\frac{\partial }{\partial b_{2N-2}}\text{log}\;P\left(Y\right)\right)\prime ,$$

because

(7) $$\frac{\partial }{\partial b_{i}}\text{log}\;P\left(Y\right)=\sum_{c=1}^{C}\frac{\partial }{\partial b_{i}}\;\text{log}\;P\left(Y_{c}\right)$$

and

(8) $$\begin{array}{c} \frac{\partial }{\partial b_{i}}\text{log}\;P\left(Y_{c}\right)=\frac{\partial }{\partial b_{i}}\text{log}\;\left(\sum_{r=1}^{R}\left[p_{\mathrm{irc}}^{\prime }q_{\mathrm{irc}}\right]P\left(\gamma_{r}\right)\right)\\ =\frac{\sum_{r=1}^{R}\frac{\partial }{\partial b_{i}}\left[p_{\mathrm{irc}}^{\prime }q_{\mathrm{irc}}\right]P\left(\gamma_{r}\right)}{\sum_{r=1}^{R}\left[p_{\mathrm{irc}}^{\prime }q_{\mathrm{irc}}\right]P\left(\gamma_{r}\right)}\\ =\frac{\sum_{r=1}^{R}\left[p_{\mathrm{irc}}^{\prime }\frac{\partial }{\partial b_{i}}q_{\mathrm{irc}}\right]P\left(\gamma_{r}\right)}{\sum_{r=1}^{R}\left[p_{\mathrm{irc}}^{\prime }q_{\mathrm{irc}}\right]P\left(\gamma_{r}\right)}\\ =\frac{\sum_{r=1}^{R}\gamma_{r}\left[p_{\mathrm{irc}}^{\prime }Q\prime q_{\mathrm{irc}}\right]P\left(\gamma_{r}\right)}{\sum_{r=1}^{R}\left[p_{\mathrm{irc}}^{\prime }q_{\mathrm{irc}}\right]P\left(\gamma_{r}\right)}, \end{array}$$

or, more intuitively, all elements in the gradient are simple weighted sums of weighted inner products involving $p_{\mathrm{irc}}$ and $q_{\mathrm{irc}}$.

### 2.1 Computing the pre-order partial likelihood vectors

For a node *i* with parent *k* and sibling *j*, Algorithm 1 outlines our approach to massively parallelize the computation of Equation (4) across all *r* and *c* simultaneously on a GPU, with each (*r*, *c*, *s*)-entry processed in its own short-lived thread. This algorithm builds on the post-order partial likelihood algorithm of Suchard and Rambaut (2009) with several important differences. First, lines 7–10 and 13–16 split the matrix-vector multiplications into two serial operations to satisfy the dependencies of Equation (4), which necessitates calculating an element-wise product before the final matrix-vector multiplication with $P^{\left(r\right)}\left(b_{i}\right)\prime$. Threads within a thread-block start executing operations on $P^{\left(r\right)}\left(b_{i}\right)\prime$ only after completing operations involving $P^{\left(r\right)}\left(b_{j}\right)$. While this reduces instruction-level parallelism, it removes the burden of storing both matrices in shared memory (ShM) simultaneously. This is especially important for transition matrices with a large state-space size in which case storing all the entries of $P^{\left(r\right)}\left(b_{j}\right)$ would overflow ShM. The second important difference is that Equation (4) requires the transpose of $P^{\left(r\right)}\left(b_{i}\right)$. When the state-space size is small as is the case for nucleotide models with S=4, we can calculate the transpose of the matrix while reading it from global memory (GM) and comfortably hold all 16 elements of the matrix in ShM. However, for models with a large state-space size such as codon models with *S *=* *61, all *S*^2^ transition probabilities do not fit in ShM simultaneously. For such cases, we also develop a ‘matrixTranspose’ kernel to calculate the transpose of $P^{\left(r\right)}\left(b_{i}\right)$ in a parallelized manner using the GPU according to Algorithm S1 (Supplementary Fig. S4). The resulting transposed matrix is stored in GM and subsequently used to calculate the pre-order partial likelihood vector $q_{\mathrm{irc}}$.

Algorithm 1. GPU-based parallel computation of pre-order partial likelihood vectors

1: **define** COLUMN_BLOCK_SIZE (CBS) = number of columns processed in parallel per thread-block

2: **define** PEELING_BLOCK_SIZE (PBS) = number of states processed in parallel per inner-loop

3: **for all** thread-blocks (rate category r=1,…,R and column-block B=1,…,⌈C/CBS⌉) **in parallel do**

4:  **for all** threads in thread-block (column c=1+(B−1)×CBS,…,B×CBS and state s=1,…,S) **in parallel do**

5:   **prefetch** post-order partial likelihood elements $p_{\mathrm{jrcs}}$ and pre-order partial likelihood elements $q_{\mathrm{krcs}}$ for CBS columns (reused by all threads in thread-block) where node *k* is parent to node *i* and its sibling node *j*.

6:   **initialize**ϕ ← 0

7:   **for**t=1,…,S in PBS-sized **parallel** chunks **do**

8:    **prefetch** transition probability elements $P_{st}^{\left(r\right)}\left(b_{j}\right)$ for PBS states (reused by all threads in the thread-block) and **synchronize**

9:      **increment**$\phi \leftarrow \phi +P_{st}^{\left(r\right)}\left(b_{j}\right)\times p_{\mathrm{jrct}}$

10:   **end for**

11:   **form** component-wise product ${\tilde{q}}_{\mathrm{krcs}}\leftarrow q_{\mathrm{krcs}}\times \phi$

12:   **initialize**ω ← 0

13:   **for**t=1,…,S in PBS-sized **parallel** chunks **do**

14:    **prefetch** (transposed) transition probability $P_{ts}^{\left(r\right)}\left(b_{i}\right)$ for PBS states (reused by all threads in the thread-block) and **synchronize**

15:       **increment**$\omega \leftarrow \omega +{\tilde{q}}_{\mathrm{krct}}\times P_{ts}^{\left(r\right)}\left(b_{i}\right)$

16:   **end for**

17:   **return** *ω* as $q_{\mathrm{ircs}}$

18:  **end for**

19: **end for**

**Figure 1.**
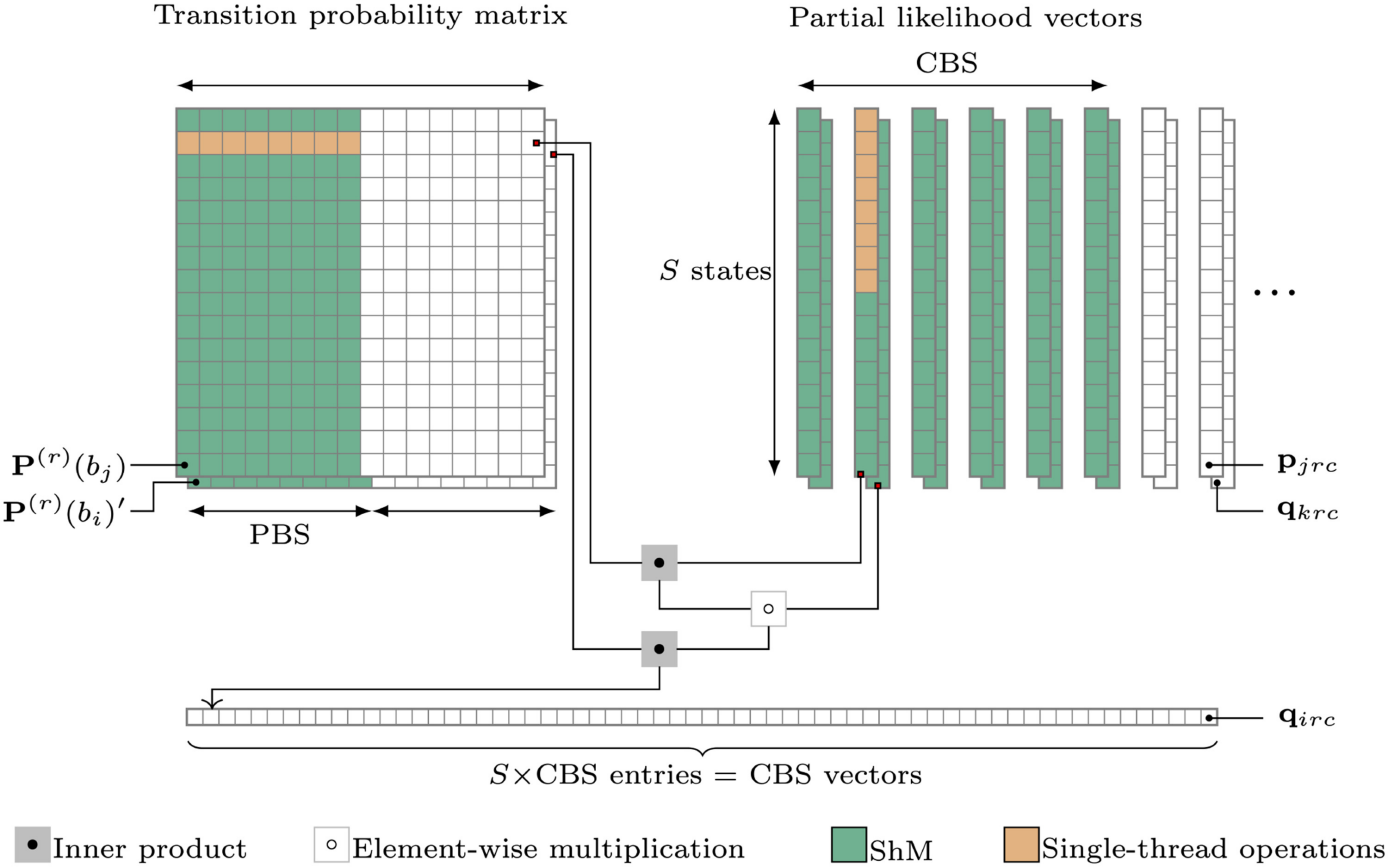
Parallel thread-block design to compute pre-order partial likelihood vectors $q_{\mathrm{irc}}$. One block evaluates column block size (CBS)×S entries in parallel and prefetches pruning block size (PBS)×S transition probability entries at a time within an inner serial loop (Algorithm 1: Lines 7–10 and 13–16). Entries fetched from global memory (GM) into shared memory (ShM) are indicated in green and an instance of a single-thread operation is shown in orange

Per thread, a surprisingly small portion of the code is dedicated to actually compute $q_{\mathrm{irc}}$. Most of the work involves efficiently fetching the transition probabilities and pre- and post-order partial likelihood vectors from GM into ShM to be reused by threads within a block. Figure 1 outlines the parallel thread-block design for Algorithm 1. We observe in Equation (4) that all *S* partials in $q_{\mathrm{irc}}$ for a column *c* under a rate class *r*, depend on the same partial likelihood vectors $q_{\mathrm{krc}}$ and $p_{\mathrm{jrc}}$. Consequentially, we construct R×⌈C/CBS⌉ thread blocks, where ⌈.⌉ is the ceiling function and column-block size (CBS) is a design constant that controls the number of columns processed in a block. Each thread-block shares S×CBS threads that correspond to all *S* states for CBS columns. All S×CBS threads within a block cooperatively fetch *S*-lengthed vectors $q_{\mathrm{krc}}$ and $p_{\mathrm{krc}}$ for CBS columns. Equation (4) also shows that under a rate class *r*, $q_{\mathrm{irc}}$ for all columns c∈C depends on the same finite-time transition probability matrices, $P^{\left(r\right)}\left(b_{i}\right)$ and $P^{\left(r\right)}\left(b_{j}\right)$. Hence, we use *S* threads to fetch columns from each of these matrices which are then reused by all threads in the thread-block. Each thread-block computes CBS pre-order partial likelihood vectors each with *S* partials.

To coalesce GM transactions and efficiently utilize the available memory bandwidth, we ensure that consecutive threads within a block attempt to access consecutive memory addresses only in multiples of 16 values at a time. For models with a state size *S* that is not a multiple of 16, we embed the transition matrices and the partial likelihood vectors into a larger space by adding zeroes as extra entries. This approach is called ‘padding’ and ensures optimal utilization of the memory bandwidth. For example, for codon models with *S *=* *61, we pad the *S* with three zero entries yielding a state-space size of 64. For nucleotide models with *S *=* *4, each thread simply processes four columns of the alignment instead of one.

Since each thread-block can have up to 512 threads (or 1024 threads depending on the hardware), we set CBS to be as large as possible such that S×CBS≤512 without overflowing ShM on the device. For nucleotide models, we set CBS=16 with each thread processing 4 columns. An additional complication arises for models with large state-space sizes such as codon models wherein all *S*^2^ transition probabilities do not fit in ShM. In such cases, *S* threads within a thread-block cooperatively fetch columns of the matrix in peeling-block size (PBS) length chunks from GM into ShM. Thus, for codon models, we set CBS=8 along with an additional design constant, PBS=4. To ensure that the GM reads of the matrix columns are coalesced, we exploit a column-wise flattened representation of the finite-time transition probability matrices which differs from the standard row-wise representation in modern computing. While newer GPUs have larger ShM available and can accommodate larger values of PBS and CBS, we set these design constraints to ensure compatibility across a broad range of devices.

### 2.2 Computing the gradient of the log-likelihood wrt all branch-specific parameters

Algorithm 2 outlines our implementation of Equation (8) to calculate the column-specific contribution to the gradient of the log-likelihood wrt all BLS parameters. To do this, we reuse the pre-order partial likelihood vectors calculated using Algorithm 1 and the post-order partial likelihood vectors calculated using the algorithm described in Suchard and Rambaut (2009). When a post-order partial likelihood vector $p_{\mathrm{irc}}$ points to a tip node and *Y_ic_* is directly observed, researchers often store this vector in a compressed format that entails a single integer (or smaller) filled with the character state to reduce memory access demands. So, for the tip nodes we simply read in the integer representation of the character state and form $p_{\mathrm{irc}}$ on-the-fly such that $p_{\mathrm{ircs}}=1$ if $Y_{ic}=s$ and 0 for all other character states. In case of missing or ambiguous data, we assign $p_{\mathrm{ircs}}=1$ for the set of possible character states.

The vector-vector multiplications needed for Equation (8) are split into two serial operations in lines 7–19 and 20 –23. Lines 7–19 perform element-wise multiplications involving $p_{\mathrm{irc}}$ and $q_{\mathrm{irc}}$ serially across rate categories. Within this section of the algorithm, lines 13–16 also perform the matrix-vector multiplication of the infinitesimal rate matrix $Q^{\left(r\right)}$ and $q_{\mathrm{irc}}$. Taken together, lines 7–19 yield *S* state-specific entries for the numerator $\phi_{c}$ and the denominator *ω_c_*. Lines 20–23 reduce these state-specific entries in parallel to calculate the column-specific contribution to the gradient wrt a BLS parameter $\frac{\partial }{\partial b_{i}}\text{log}\;P\left(Y_{c}\right)$.

Algorithm 2. GPU-based parallel computation of the gradient of the log-likelihood wrt all branch-specific parameters

1: **define** COLUMN_BLOCK_SIZE (CBS) = number of columns processed in parallel per thread-block

2: **define** PEELING_BLOCK_SIZE (PBS) = number of states processed in parallel per inner-loop

3: **for all** thread-blocks (node i=1,…,2N−2 and column-block CB=1,…,⌈C/CBS⌉) **in parallel do**

4:  **for** *R* threads in thread-block (rate r=1,…,R) **in parallel do**

5:   **prefetch** weight $P\left(\gamma_{r}\right)$ (reused by all threads in thread-block)

6:  **end for**

7:  **for all** threads in thread-block (column c=1+(B−1)×CBS,…,B×CBS and state s=1,…,S) **in parallel do**

8:   **initialize**$\phi_{cs}\leftarrow 0,\;\omega_{cs}\leftarrow 0$

9:   **for**r=1,…,R**in series do**

10:    **prefetch** post-order partial likelihood elements $p_{\mathrm{ircs}}$ and pre-order partial likelihood elements $q_{\mathrm{ircs}}$ for CBS columns (reused by all threads in thread-block) and **synchronize**

11:    **increment**$\omega_{cs}\leftarrow \omega_{cs}+p_{\mathrm{ircs}}\times q_{\mathrm{ircs}}\times P\left(\gamma_{r}\right)$

12:    **initialize**δ←0

13:    **for**t=1,…,S in PBS-sized **parallel** chunks **do**

14:     **prefetch** infinitesimal rate elements $Q_{st}^{\left(r\right)}$ for PBS states (reused by all threads in the thread-block) and **synchronize**

15:     **increment**$\delta \leftarrow \delta +Q_{st}^{\left(r\right)}\times q_{\mathrm{irct}}$

16:    **end for**

17:    **increment**$\phi_{cs}\leftarrow \phi_{cs}+p_{\mathrm{ircs}}\times \delta \times P(\gamma_{r})$

18:   **end for**

19:  **end for**

20:  **for** CBS tasks (c=1,…,CBS) with *S* threads (s=1,…,S) each **in parallel do**

21:    **reduce**$\phi_{c}\leftarrow \sum_{s=1}^{S}\phi_{cs}$ and $\omega_{c}\leftarrow \sum_{s=1}^{S}\omega_{cs}$

22:      **return**$\phi_{c}/\omega_{c}$ as column-specific contribution $\frac{\partial }{\partial b_{i}}\text{log}\;P\left(Y_{c}\right)$

23:  **end for**

24: **end for**

**Figure 2.**
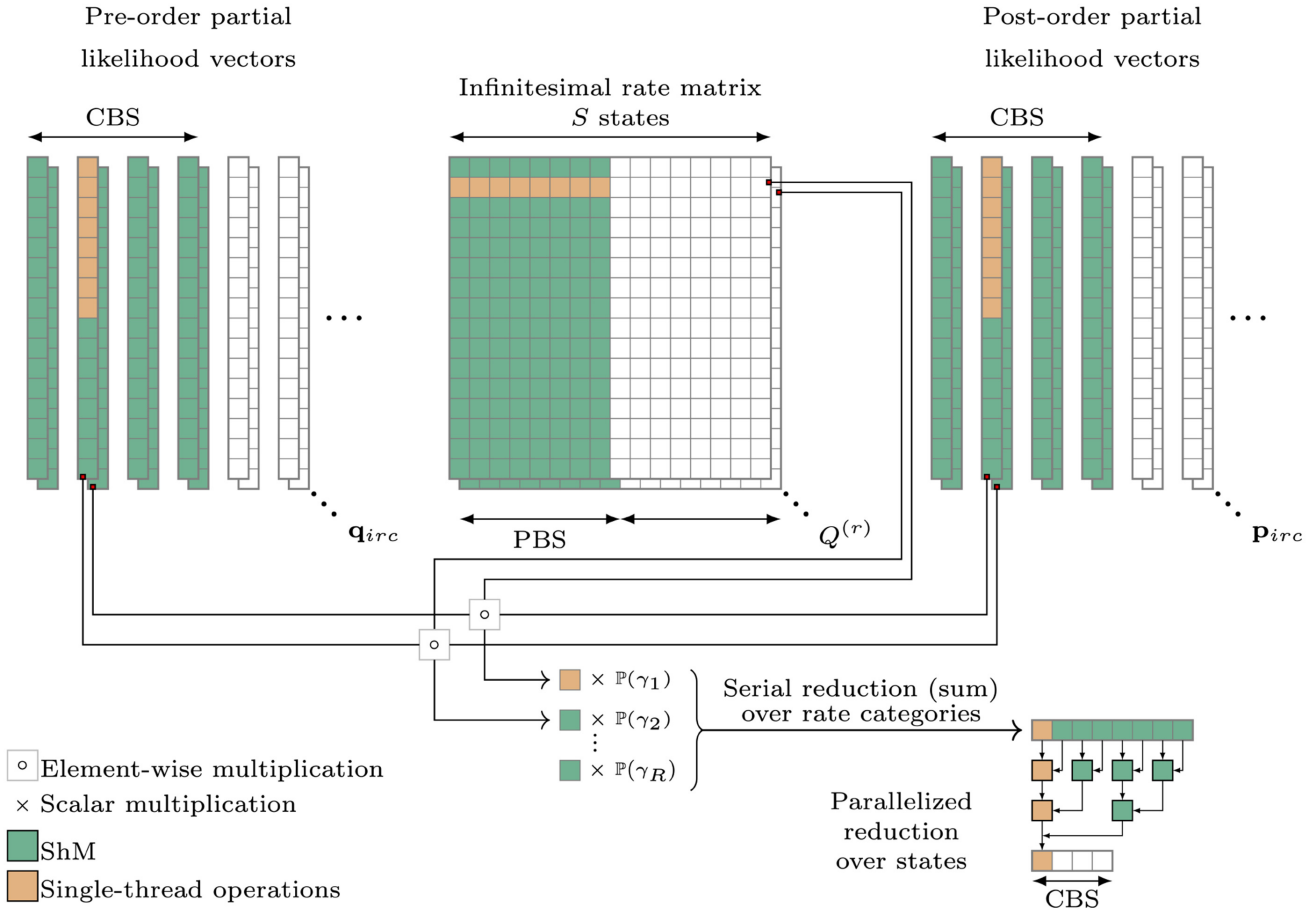
Parallel thread-block design to calculate the column-specific contributions to the gradient of the log-likelihood wrt all BLS parameters for all columns *C*. One block evaluates the column-specific contribution to the gradient wrt a BLS parameter $\frac{\partial }{\partial b_{i}}\text{log}\;P\left(Y_{c}\right)$ for CBS columns by prefetching S×CBS entries from the pre- and post-order partial likelihood vectors $q_{\mathrm{irc}}$ and $p_{\mathrm{irc}}$ in parallel (Algorithm 2: Line 10) and S×PBS entries at a time from $Q^{\left(r\right)}$ within an inner serial loop (Algorithm 2: Lines 13–16). Each block performs a serial reduction over rate categories (Algorithm 2: Lines 7–19) and a parallelized reduction over states (Algorithms 2: Lines 20–23). Entries fetched from global memory (GM) into shared memory (ShM) are indicated in green and an instance of a single-thread operation is shown in orange

The CPU implementation of Equation (8) only parallelizes calculations across conditionally independent blocks of the sequence alignment, but Algorithm 2 takes advantage of the GPU to introduce a great deal of fine-scale parallelization across nodes and columns. Similar to Algorithm 1, most of the work per thread involves effectively caching values in ShM to be reused by the threads within a thread-block. Figure 2 outlines the parallel thread-block design for Algorithm 2. Since the weights $P\left(\gamma_{r}\right)$ of the rate categories are the same for all columns, we use *R* threads within a block to fetch these weights into ShM. Equation (8) shows that calculating $\frac{\partial }{\partial b_{i}}\text{log}\;P\left(Y_{c}\right)$ for node *i* and column *c* involves taking a weighted sum of rate-specific entries. Each rate-specific entry depends on the same *S* partial likelihoods from each of the partial likelihood vectors $q_{\mathrm{irc}}$ and $p_{\mathrm{irc}}$. Based on this observation, we construct (2N−2)×⌈C/CBS⌉ thread-blocks with each thread-block sharing S×CBS threads. All S×CBS threads within a block concurrently fetch *S*-lengthed vectors $q_{\mathrm{irc}}$ and $p_{\mathrm{irc}}$ for CBS columns from GM into ShM. We also observe that the rate-specific entries for all columns depend on the same infinitesimal rate matrix $Q^{\left(r\right)}$. This allows us to use *S* threads within a block to fetch columns from the rate matrix in PBS chunks from GM into ShM as described previously in Algorithm 1. Each thread-block computes $\frac{\partial }{\partial b_{i}}\text{log}\;P\left(Y_{c}\right)$ for one node and CBS columns.

Since columns in the sequence alignment are assumed to be independent, arising from conditionally independent CTMCs acting along each branch, we can calculate $\frac{\partial }{\partial b_{i}}\text{log}\;P\left(Y\right)$ by reducing the partial derivatives across all columns *C* according to Equation (7). We wrote a ‘nodeSiteReduction’ kernel to perform this reduction in parallel with each thread-block reading in 128 column-specific contributions to the gradient. For certain models such as those that assume a strict molecular clock, it might be more convenient to further reduce the partial derivatives across a set of branches and report a single value. We currently perform this final reduction on the CPU to maintain the ability to specify any desired set of branches. The design constants CBS and PBS for Algorithm 2 are the same as Algorithm 1 with CBS=16 for nucleotide models and CBS=8PBS=4 for codon models.

Our current model assumes $Q^{\left(r\right)}=\gamma_{r}Q$, but we have left the algorithm for the more general case when $Q^{\left(r\right)}$ can vary arbitrarily across rate categories. Without this generalization, we could reduce some memory transactions by reading $\gamma_{1},\ldots ,\gamma_{R}$ and **Q** once and forming $Q^{\left(r\right)}=\gamma_{r}Q$ on-the-fly. Another limitation of Algorithm 2 arises when R>S×CBS even though R≤10 is adequate for most common phylogenetic analyses (Jia *et al.* 2014). In such a case, lines 7–19 in Algorithm 2 can be executed serially on chunks of rate categories that can be controlled by introducing a new, rate-block-size design constant.

Finally, it is worth noting that direct recursive application of Equation (2) to evaluate P(Y) often generates floating-point underflow. To side-step this issue, one can instead manipulate re-scaled post-order likelihood vectors ${\hat{p}}_{\mathrm{irc}}=p_{\mathrm{irc}}/M_{ic}$ where $M_{ic}$ are node- and column-specific scalars selected to keep the elements of ${\hat{p}}_{\mathrm{irc}}$ bounded. Fortuitously, replacing $p_{\mathrm{irc}}$ with ${\hat{p}}_{\mathrm{irc}}$ in Equations (4) and (8) does not change the evaluation of Equation (8) since the constants in the numerator and denominator cancel. As a consequence, one can directly use the rescaled likelihood vectors to evaluate the gradient, while avoiding underflow. Supplementary Section S1.2 provides further details.

## 3 Results

We use three datasets to illustrate the performance gains afforded by the algorithms presented in this paper: (i) a dengue virus dataset of 997 genomes with 6869 unique nucleotide site patterns across 10 genes and 3343 unique site patterns when translated into a 61 state universal codon model, (ii) a carnivores dataset of 62 genomes with 5565 unique nucleotide site patterns and 3600 unique site patterns when translated into a 61 state vertebrate mitochondrial codon model, and (iii) a yeast dataset of 49 genomes with 12 878 unique nucleotide site patterns and 22 151 unique site patterns when translated into a 61 state universal codon model. To provide comprehensive estimates of performance gains, we use three systems with varied technical specifications. System 1 is equipped with a 10-core 3.3 GHz Intel Xeon W-2155 processor, 32 GB 2.6 GHz DDR4 RAM and an NVIDIA Quadro GV100 GPU with 5120 cores running at 1.1 GHz and 32 GB global memory. System 2 is equipped with a 20-core 2.2 GHz Intel Xeon E5-2698 processor, 512 GB 2.6 GHz DDR4 RAM and an NVIDIA Tesla V100 GPU with 10 240 cores running at 1.53 GHz and 32 GB global memory. System 3 is equipped with a 48-core 2.3 GHz AMD EPYC 7642 Processor, 512 GB DDR4 RAM and an AMD MI50 GPU with 3840 cores running at 1.73 GHz and 32 GB global memory.

**Figure 3.**
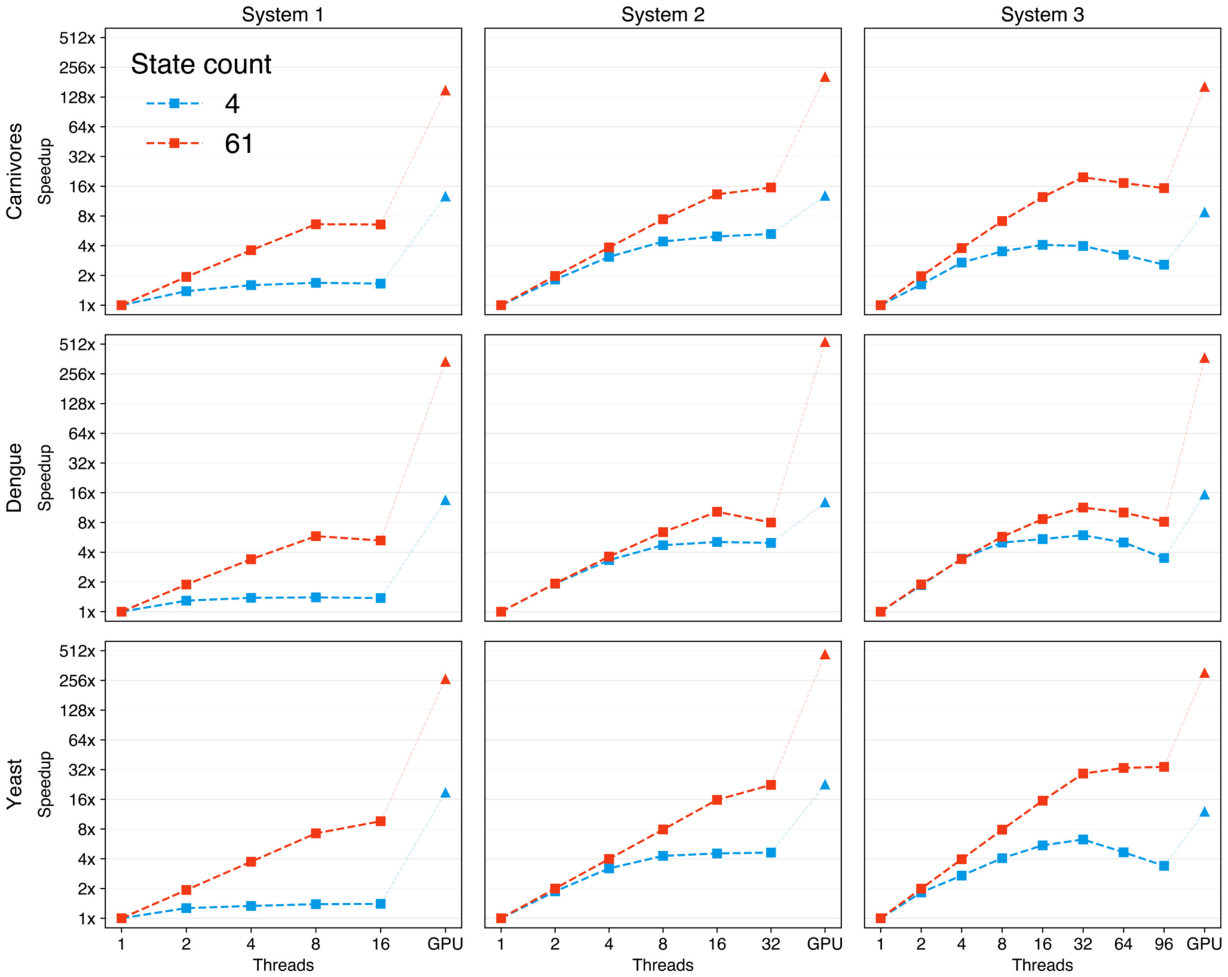
Speedup of GPU and multi-core CPU instances over a single CPU thread for five MCMC iterations to infer branch-specific evolutionary rates for a GTR model with state-space size of 4 and a Yang codon model with a state-space size of 61. The comparisons are reported for three datasets on three different systems. Speedup factors are on a log-scale

For each dataset, we infer branch-specific evolutionary rates given fixed trees from a Bayesian analysis for two substitution models, the general time reversible (GTR) nucleotide model (Tavaré and Miura 1986) including discrete gamma-distributed rate variation with four categories and the Yang codon model (Yang *et al.* 2000). We perform this analysis on each of the three systems and measure the wall-time of five iterations of the MCMC using HMC as described in Fisher *et al.* (2020) and implemented in the Bayesian phylogenetic reconstruction software package BEAST (Suchard *et al.* 2018). We report the corresponding speedup of multi-threaded CPU and GPU instances over a single-threaded CPU in Fig. 3. For the GTR model, we see that performance on the CPU reaches saturation at 32 threads on systems 2 and 3 with a near 4-fold speedup as compared to single-core performance on all three datasets. On system 1 which is equipped with a less powerful CPU, we see that the multi-threaded CPU implementation offers more modest speedups of <2-fold. On system 3 which has a CPU with 48 cores, we see that increasing the number of threads over 32–64 and 96 results in longer wall-times. Our algorithms that utilize the GPU offer a higher speedup of near 16-fold on all three systems.

We consistently see higher performance gains for the Yang codon model with a state-space size of 61 compared to the 4-state GTR model on both the CPU and GPU. For the Yang codon model, the performance of the CPU implementation reaches saturation at 8, 16, and 32 threads on systems 1, 2, and 3 respectively. On the CPU, we see a maximum speedup of near 8-fold on system 1 and near 16-fold on systems 2 and 3 for all three datasets with the exception of the yeast dataset on system 3. The yeast dataset yields a much higher number of unique site patterns when translated into a 61-state codon model compared to the other two datasets and the powerful 48-core CPU on system 3 offers a maximum speedup of near 32-fold. As we previously observed for the GTR model, using >32 CPU threads on system 3 results in a decrease in performance. The GPU for the Yang codon model offers speedups of >128-fold on all three systems, which far exceeds the performance gain that can be obtained from a multi-threaded CPU.

To determine whether increasing the state-space size beyond 61 would result in higher performance gains on the GPU, we also inferred branch-specific evolutionary rates for the yeast dataset using a Markov-modulated model (MMM) composed of two Yang codon models yielding a combined state-space size of 122. The speedups for the MMM model are very similar to the Yang codon model, showing that the resources on the GPU are maximally utilized at a state-space size of 61 (Supplementary Fig. S3).

Further, we report the performance gains specifically for calculating the pre-order partial likelihood vectors and the gradient of the log-likelihood wrt all BLS parameters in Table 1. We see that for the GTR model, the GPU implementation offers over 19-fold improvement for calculating the pre-order partial likelihood vectors and over 7-fold improvement for calculating the gradient. The GPU implementation for the Yang codon model with a larger state-size of 61 shows a much higher performance gain with over 170-fold improvement for calculating the pre-order partial likelihood vectors and over 225-fold improvement for calculating the gradient. For a complete overview, we report both the achieved speedup and the percentage of wall-time consumed for all the parallelizable functions in Supplementary Table S1.

**Table 1.** Speedup in calculating pre-order partial likelihood vectors and the gradient of the log-likelihood for a GTR model and a Yang codon model on the GPU relative to a single-threaded CPU.

State count	Dataset	Pre-order traversal			Gradient calculation		
		Sys 1	Sys 2	Sys 3	Sys 1	Sys 2	Sys 3
4	Carnivores	23	27	19	23	32	16
	Dengue	29	31	24	7	8	14
	Yeast	31	39	24	19	35	18
61	Carnivores	231	225	216	225	402	284
	Dengue	170	195	179	429	819	586
	Yeast	312	544	290	446	812	588

Since our algorithm for the pre-order traversal builds on the post-order traversal algorithm described in Suchard and Rambaut (2009), we measured the total time expended by the relevant kernels on the GPU while inferring branch-specific evolutionary rates under the Yang codon model using the carnivores dataset (Supplementary Table S2). It is important to note that this metric differs from the previously cited wall-time. The total time used by a given kernel on a GPU includes the durations of individual kernel calls, some of which may be executed concurrently on the GPU. This enables a precise comparison between the massively parallel algorithms for the post- and pre-order traversal. We observe that the average time per function call for the post-order traversal (126 096 ns) is faster than the pre-order traversal (177 096 ns). The pre-order kernel is also called nearly two times more than the post-order kernel since pre-order partial likelihoods must be calculated at the tip nodes, whereas the *Y_ic_* is directly observed for the post-order partial likelihoods at the tip nodes. This latter point also explains why the small speed-difference is unsurprising. In spite of the reduced burden on ShM, the pre-order traversal requires larger GM transactions than the post-order traversal immediately above and at the tip nodes; when *Y_ic_* is observed, the post-order partial likelihoods are sparse, while the pre-order partial likelihoods are always dense. Overall, the pre-order kernel takes 44.62% of the total wall-time while the post-order kernel takes 17.21%. In addition to Algorithms 1 and 2, we also implemented two additional kernels, namely, the matrixTranspose kernel to transpose transition matrices for models with large state-space sizes and the nodeSiteReduction kernel to reduce the column-specific contributions to the gradient across columns. Both these kernels require very little execution time, with the matrixTranspose and nodeSiteReduction kernels taking roughly 0.26% and 0.04% of the total wall-time, respectively (Supplementary Table S2).

**Figure 4.**
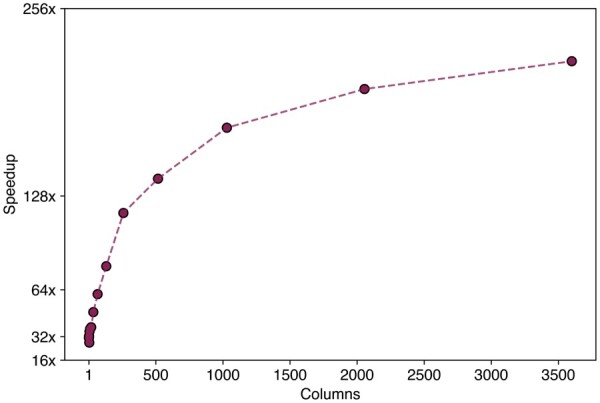
Speedup on the GPU relative to a single threaded CPU scaled by the number of unique alignment columns *C* using the codon-alignment of the carnivores dataset. Speedup factors are on the log-scale

Apart from state-space size *S*, another dimension that influences the effectiveness of parallelization on GPUs is the number of unique site patterns or alignment columns *C*. For a small number of columns, the overhead of caching values in ShM might outweigh the performance gain afforded by the parallelization of the numerical calculations. As the number of columns increases, we expect performance to increase until it reaches saturation when the resources on the GPU are fully utilized. To measure how performance scales with the number of columns, we truncated the codon alignment of the carnivores dataset to obtain an increasing number of unique site patterns and report the associated speedup on the GPU over a single-threaded CPU instance in Fig. 4. Even for a single column, we observe a speedup of nearly 31-fold with performance reaching saturation at *C *=* *1, 024 indicating the maximal utilization of the resources on the GPU at that point followed by marginal increases in performance as more columns are added.

## 4 Example

To demonstrate the utility of the algorithms presented in this paper, we infer the date of the first introduction of West Nile virus into the United States under the Yang codon model with branch-specific evolutionary rates. West Nile virus is a mosquito-borne RNA virus that was first detected in the United States in New York City in August 1999 [Centers for Disease Control and Prevention (CDC) 1999]. Since its first detection on the East Coast in 1999, the virus spread westward across the continental United States and was first detected on the West Coast in California in November 2003 (Reisen *et al.* 2004). The virus has caused over 52 000 cases and over 2400 deaths as of 2020, making it the leading cause of domestically acquired arbovirus disease in the continental United States (Soto *et al.* 2022).

**Figure 5.**
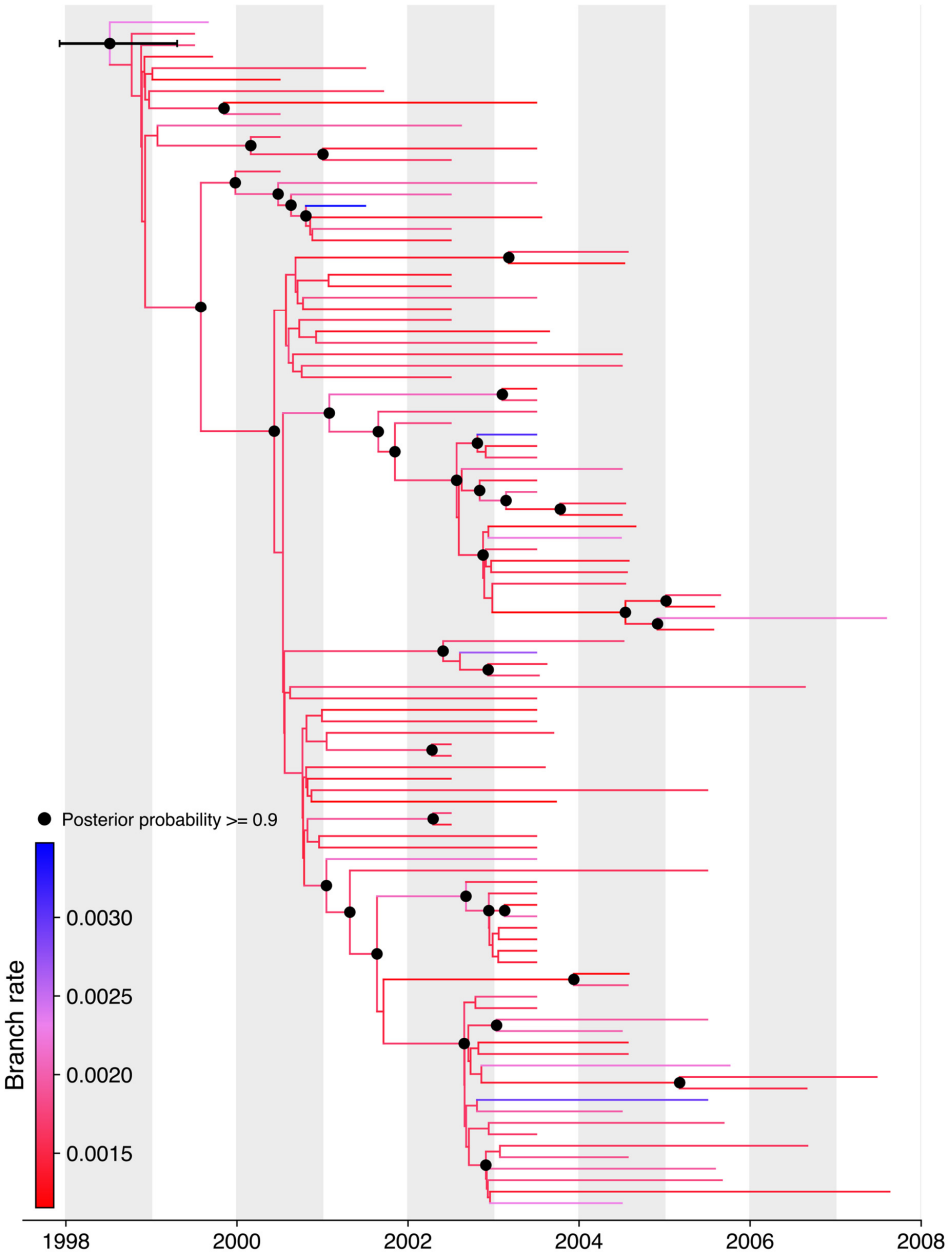
Reconstructed codon-based maximum clade credibility (MCC) tree of 104 West Nile virus genomes sampled in the continental United States between 1999 and 2007

Here, we use a dataset of 104 full viral genomes collected in the continental United States between 1999 and 2007 (Pybus *et al.* 2012). Each genome encodes for a single polyprotein precursor that is post translationally cleaved into three structural and seven nonstructural proteins (Brinton 2002). When translated into a 61-state universal codon model, this alignment yields 1126 unique site patterns. We infer the age of the root of these genomes from a Bayesian analysis under the Yang codon model with a 4-class discrete-Γ model for site rate variation (Yang 1996), an uncorrelated relaxed clock model (Drummond *et al.* 2006) and a skyline nonparametric coalescent prior (Drummond *et al.* 2005) on the unknown tree. Figure 5 displays the maximum clade credibility (MCC) tree inferred using BEAST with HMC over the branch-specific rate scalars, node heights, and the parameters of the skyline population model. The default transition kernels were used over the remaining random parameters including the unknown tree and codon model parameters. We ran this analysis for 10 million MCMC iterations with the first 10% of iterations discarded as burn-in. The effective sample size (ESS) for all scientifically relevant parameters was above 200. Convergence of the chain and ESS of parameters were assessed using Tracer (Rambaut *et al.* 2018) and the MCC tree was constructed using TreeAnnotator 1.10 and visualized using baltic (https://github.com/evogytis/baltic).

Table 2 reports the marginal posterior estimates for the age of the root, the transition: transversion ratio *κ*, and the nonsynonymous: synonymous rate ratio *ω*.

**Table 2.** Parameter estimates of Yang codon model for 104 West Nile virus genomes sampled in the continental United States between 1999 and 2007.

Parameter	Posterior median	95% Bayesian credible interval
Age of root	1998.58	(1997.72–1999.10)
Transition: transversion rate *κ*	11.34	(9.33–13.55)
*dN/dS* ratio *ω*	0.14	(0.12–0.16)

The posterior median estimate for the age of the root was 1 August 1998 (95% highest posterior density [HPD] interval: [September 1997, February 1999]) that stands in line with previous studies that inferred the age of the root from a Bayesian analysis under a nucleotide model (Pybus *et al.* 2012). This result is also consistent with prior research that suggested a similar introduction time based on circumstantial evidence (Lanciotti *et al.* 1999). As reported previously in Añez *et al.* (2013), the virus has been subjected to strong purifying selection since its introduction as evidenced by a posterior median *dN*/*dS* ratio *ω* estimate of 0.14 (95% HPD: [0.12, 0.16]). The consistency with prior findings from the literature also validates the estimates from our GPU-based algorithms. We performed this analysis on a desktop equipped with a 20-core 2.2 GHz Intel Xeon E5-2698 processor, 512 GB 2.6 GHz DDR4 RAM and an Nvidia Tesla V100 GPU with 10 240 cores running at 1.53 GHz and 32 GB global memory. We estimated that this analysis would take ∼1500 days on a CPU based on executing 26 000 iterations which took ∼3600 h/million iterations. Using our algorithms on the GPU, we were able to complete this analysis in just roughly 100 hours, a substantial improvement compared to the estimated runtime on a CPU.

## 5 Discussion

The algorithms presented in this paper utilize GPUs to deliver several orders of magnitude speedup over corresponding CPU implementations in calculating the pre-order partial likelihood vectors and subsequently evaluate the gradient of the data log-likelihood wrt all BLS parameters. Multi-core CPUs can provide an increase in performance but eventually reach a saturation point beyond which increasing the number of threads results in a decrease in performance. The algorithms presented in this paper are memory-bound on both the CPU and the GPU but GPUs typically offer a substantially larger memory bandwidth than CPUs. For instance, on system 3, the 48-core AMD EPYC 7642 processor has a maximum memory bandwidth of 200 GB/s (AMD 2019) while the AMD MI50 GPU boasts a maximum memory bandwidth of 1 Tb/s (AMD 2018). Consequently, using multiple cores on the CPU will result in the exhaustion of the available memory bandwidth, and ultimately lead to a decrease in performance. The larger memory bandwidth and the availability of fine-scale parallelization on GPUs, enable our algorithms to achieve performance gains that cannot be attained trivially by adding more threads available on a CPU. The improvement in performance for nucleotide models is noteworthy, but becomes even more remarkable when applied to models with a large state-space size such as codon models. This performance gain enables Bayesian phylogenetic analyses to infer branch-specific parameters using gradient-based samplers like HMC, as showcased by the example of dating the first introduction of West Nile virus into the continental United States. We also note that while libraries such as the Phylogenetic Likelihood Library (Flouri *et al.* 2015) provide high performance CPU implementations of likelihood calculations required for phylogenetics, to the best of our knowledge, no other existing software implements algorithms to calculate the gradient wrt BLS parameters similar to the ones presented in this study. There also exist general-purpose machine learning libraries such as PyTorch and TensorFlow that use automatic differentiation (AD) to evaluate the gradients of arbitrary models. Fourment *et al.* (2023) use the CPU versions of TensorFlow, PyTorch, JAX and Stan to evaluate the gradient of phylogenetic likelihood functions and benchmark these implementations against the analytical gradients used in the CPU implementation of the algorithms presented in this study. The authors find that the analytical gradients are at least eight times faster than the fastest AD implementation with varied performance across different AD implementations. Additionally, their preliminary experiments with GPU-acceleration in these libraries does not yield significant improvements in speed, underscoring the importance of carefully designed GPU kernels for effective performance improvement.

Our algorithms also have implications for researchers looking to invest in hardware upgrades in order to increase computational speed in a cost-effective manner. As of January 2023, AMD Ryzen Threadripper PRO 5975WX with 64 cores (retails for around $6500 on Newegg) has the highest number of cores among commercially available CPUs but costs twice as much as an Nvidia Tesla V100 GPU (retails for around $3500 on Newegg) which has sufficient double-precision floating point performance for phylogenetic analyses. Thus, GPUs offer a much lower price-performance ratio compared to CPUs which is of consequence for any decision regarding the purchase of new systems or the upgrade of existing systems for individuals as well as high performance computing clusters at academic and nonacademic institutions. The ubiquity of cloud computing has allowed researchers to perform computationally intensive analyses without having to purchase their own hardware. Even in this scenario, the cost of a compute instance equipped with a single GPU is more cost-efficient compared to one equipped with a large number of CPU cores. For instance, on Amazon web services as of January, 2023, the on-demand pricing of p3.2xlarge with 1 Tesla V100 is $3.06/h whereas the c6g.8xlarge with 32 threads (vCPUs) costs $1.088/h. However, the speedups afforded by our algorithms using the GPU are 4- to 6-fold higher than using 32 threads on a CPU making the former more cost-efficient. Considering the ongoing decline in computing costs, GPUs are poised to remain more cost-effective than CPUs for phylogenetic analyses.

An additional key consideration when evaluating GPU and CPU performance is their respective energy efficiencies. Energy consumption on a GPU can be assessed using command line utilities such as nvidia-smi or libraries like the NVIDIA Management Library. Conversely, quantifying the energy consumption on a CPU is nontrivial, complicating direct energy efficiency comparisons between running our algorithms on a GPU versus a CPU. Here, we attempt to bound this relative efficiency by considering the maximum energy specification of the CPU and GPU used on System 2. System 2 was equipped with a 20-core 2.2 GHz Intel Xeon E5-2698 processor and an NVIDIA Tesla V100 with a maximum power consumption of 135 W and 300 W, respectively. Given that even the GPU implementation requires CPU time for initializing the phylogenetic model and issuing the relevant numerical tasks to be performed on the device, we roughly estimate the energy efficiency of running an algorithm for an equivalent duration on the GPU versus the CPU to be $\frac{135+300}{135}∼3.2$. As shown in Fig. 3, the GPU implementation for the 61-state Yang codon model is at least 13-fold faster compared to the fastest CPU implementation utilizing 32 cores, making it more energy-efficient than the CPU. For the 4-state GTR model, however, we observe a speedup of ∼ 2.5 for both the carnivores and Dengue datasets suggesting that the CPU is marginally more energy-efficient compared to the GPU in these cases. Although on the yeast dataset, we see ∼4.8 speedup on the GPU showing that for a large number of unique site patterns, a GPU could be more energy efficient even for a 4-state model. As energy efficiency is an important criterion in employing hardware like GPUs, these results will shape subsequent work aimed at optimizing the energy efficiency of the kernels, especially for the smaller 4-state models.

There remain several limitations to our current massively parallel algorithms. Among these, for *S *>* *4, we currently take as input the transpose of a transition probability matrix for the pre-order computation, and we compute this transposition via a separate matrixTranspose kernel. Currently, we compute all matrix transpositions in parallel to avoid an additional kernel launch for each pre-order evaluation. This requires storing both the original matrices and their transposes in GM. In a piece of on-going research, we are utilizing the log-likelihood gradient wrt over 20 000 branch lengths under an *S *=* *256 model. These transposed matrices require approximately 20,000×256×256×8bytes≈10GB of additional RAM in double-precision, severely restricting the range of GPUs that we can employ. Memory constraints due to too many columns can be addressed by using multiple GPUs but this is not a solution for transition matrix memory constraints. We could, however, split this operation by scheduling separate kernels across multiple command queues (or CUDA streams for NVIDIA devices) with each queue concurrently computing the transpose of a single transition matrix and the corresponding pre-order partial likelihoods. This would remove the need to store all the transition matrices and their transposes in GM simultaneously.

The CPU implementation used in this study utilizes explicit single-instruction multiple-data (SIMD) vectorization through SSE2 instructions for the 4-state GTR model while for the 61-state codon model we rely on the compiler automatically issuing vectorized FMA instructions, storing memory operands in XMM registers. There exist additional ways to improve performance on the CPU to evaluate the log-likelihood gradient. The current CPU implementation only allows concurrent execution across conditionally independent blocks of columns in the alignment but additional parallelization across nodes can also be exploited. However, it is unlikely that any additional parallelization on the CPU would lead to a speedup that is on the same scale as the improvement achieved by using GPUs.

While the algorithms in this study were presented in a Bayesian framework, they also have applications in nonlinear optimization in a maximum-likelihood framework. To make our algorithms available to the broader audience of developers working on statistical phylogenetics, we provide implementations in the open-source BEAGLE v4.0.0 library (Ayres *et al.* 2019) that uses OpenMP for multi-core CPUs, CUDA and OpenCL for GPUs.

## Supplementary Material

btae030_Supplementary_Data

## Data Availability

Complete BEAST XML files and associated scripts to reproduce the three performance study datasets and WNV example are available at https://github.com/suchard-group/parallel_gradients_supplement. The log files from the benchmarking and the results from the WNV analysis have been deposited at https://doi.org/10.5281/zenodo.7697474.

## Software availability

The algorithms described in this article have been implemented in BEAGLE v4.0.0 available at https://github.com/beagle-dev/beagle-lib/releases/tag/v4.0.0.
