# Supplementary material for: Many-core algorithms for high-dimensional gradients on phylogenetic trees
Source: Bioinformatics. 2024 Jan 18;40(2):btae030. doi: 10.1093/bioinformatics/btae030 (PMC10868298; doi:10.1093/bioinformatics/btae030)
Supplement: btae030_Supplementary_Data [file btae030_supplementary_data.pdf]

Supplementary Material

Considerations for GPU algorithms

The design of our algorithms is guided by certain aspects of GPU architecture to achieve optimal performance on the available resources. A GPU can consist of hundreds to over a thousand or so parallel processors known as streaming multiprocessors (SMs) or CUDA cores on NVIDIA devices. Each SM can execute multiple threads that are further grouped into thread-blocks with each thread-block containing up to 512 to 1024 threads as determined by hardware constraints. Within a thread-bock, groups of 16 or 32 consecutive threads, termed warps, are executed in parallel using a single instruction, multiple threads (SIMT) execution model. Functions, called kernels, are executed by thread-blocks that are indexed as a grid. Each SM has 256 KB of register memory (64,000 32-bit registers) and each thread can be allocated a maximum of 1 KB (255 registers). Registers are allocated exclusively to a single thread and cannot be accessed by other threads. In addition to register memory, all threads within a thread-block are allocated shared memory (ShM) of 32 or 64 KB as determined by hardware constraints and all thread-blocks have access to the global memory (GM) on the GPU that is typically a dynamic random-access memory device (Figure S1). ShM is located on chip and, hence, memory access can be 100- to 150-fold faster than GM transactions. Since all threads within a thread-block have access to the same ShM, each thread within a block can cooperatively fetch values from global memory and cache them in shared memory for subsequent operations. To further minimize the number of memory transactions with GM, memory access by consecutive threads within a warp or half warp, are combined or 'coalesced' into one or more 32-, 64- or 128-byte transactions based on hardware constraints. The most optimal GM access pattern is achieved when consecutive threads within a warp or half warp access consecutive memory addresses in GM which maximizes the use of the available bandwidth of each 'coalesced memory transaction'.

Once a warp executes an instruction, it has to wait for a fixed number of clock cycles, called the latency, before executing the next instruction. Typically the latency for an instruction that requires GM access is hundreds of clock cycles compared to far fewer clock cycles for ShM access. Maximum utilization of available resources on the GPU can be achieved by ensuring that there are sufficient warps queued to execute a new instruction at each clock cycle while other warps wait until their memory transactions are complete. This approach hides memory latency and motivates the fine-scale parallelization of any numerical operation executed using a GPU. In addition to having low latency ShM, synchronization between threads within the same block i.e, coordinating the execution of threads when they access the sa5me resources such as ShM or GM is inexpensive. This makes parallelization efficient for even small binary reductions of numbers which is not the case for multi-core CPUs.

Scaling partial likelihoods to mitigate underflow errors

Suchard & Rambaut (2009) describe a scaling strategy for GPUs when floating-point underflow occurs while recursively calculating the post-order partial likelihood vectors. Under this strategy, one instead recursively manipulates re-scaled post-order likelihood vectors $\hat{\mathbf{p}}_{irc} = \mathbf{p}_{irc}/M_{ic}$ where M_{ic} are node- and column-specific scalars selected to keep the elements of $\hat{\mathbf{p}}_{irc}$ bounded. A natural choice for $M_{ic} = \max_{rs} p_{ircs}$.Here we demonstrate that replacing

Algorithm S1 GPU-based parallel computation of the finite-time transition probability matrix transpose.

define MULTIPLY_BLOCK_SIZE (MBS) = number of rows and columns processed in parallel per thread-block.
for all thread-blocks (tile-row $= 1, \ldots, \lceil S/\text{MBS} \rceil$ and tile-col $= 1, \ldots, \lceil S/\text{MBS} \rceil$) **in parallel do**
    for all threads in thread-block ($s = 1, \ldots, \text{MBS}$ and $t = 1, \ldots, \text{MBS}$) **in parallel do**
        A[s][t] $\leftarrow P^{(r)}_{st}(b_i)$
        return A[t][s]
    end for
end for

$\mathbf{p}_{irc}$ with $\hat{\mathbf{p}}_{irc}$ in Equations (4) and (8) does not change the evaluation of Equation (8) since the constants in the numerator and denominator cancel. Starting with Equation (4), let

$$\hat{\mathbf{q}}_{irc} = \left[\mathbf{P}^{(r)}(b_i)\right]' \left\{\mathbf{q}_{krc} \circ \left[\mathbf{P}^{(r)}(b_j)\right] \hat{\mathbf{p}}_{jrc}\right\} = \frac{1}{M_{jc}} \mathbf{q}_{irc},$$

then substituting $\hat{\mathbf{p}}_{irc}$ and $\hat{\mathbf{q}}_{irc}$ into the final line of Equation (8) yields

$$\begin{aligned} \frac{\sum_{r=1}^{R} \gamma_r \left[\hat{\mathbf{p}}'_{irc} \mathbf{Q}' \hat{\mathbf{q}}_{irc}\right] \mathbb{P}(\gamma_r)}{\sum_{r=1}^{R} \left[\hat{\mathbf{p}}'_{irc} \hat{\mathbf{q}}_{irc}\right] \mathbb{P}(\gamma_r)} &= \frac{\cancel{\frac{1}{M_{ic}}} \cancel{\frac{1}{M_{jc}}} \sum_{r=1}^{R} \gamma_r \left[\mathbf{p}'_{irc} \mathbf{Q}' \mathbf{q}_{irc}\right] \mathbb{P}(\gamma_r)}{\cancel{\frac{1}{M_{ic}}} \cancel{\frac{1}{M_{jc}}} \sum_{r=1}^{R} \left[\mathbf{p}'_{irc} \mathbf{q}_{irc}\right] \mathbb{P}(\gamma_r)} \\ &= \frac{\sum_{r=1}^{R} \gamma_r \left[\mathbf{p}'_{irc} \mathbf{Q}' \mathbf{q}_{irc}\right] \mathbb{P}(\gamma_r)}{\sum_{r=1}^{R} \left[\mathbf{p}'_{irc} \mathbf{q}_{irc}\right] \mathbb{P}(\gamma_r)} \\ &= \frac{\partial}{\partial b_i} \log \mathbb{P}(\mathbf{Y}_c)\,. \end{aligned}$$

Of note, further rescaling of the pre-order likelihood vectors $\mathbf{q}_{irc}$ (or, similarly, $\hat{\mathbf{q}}_{irc}$) during their recursive computation via Equation (4) is also possible. A reasonable choice is $\tilde{\mathbf{q}}_{irc} = \mathbf{q}_{irc}/Q_{ic}$ where scalar $Q_{ic} = \max_{rs} q_{ircs}$. The proof above easily generalizes with this additional rescaling. Across the data examples we have explored using 64-bit floating-point operations, such an additional rescaling remains unnecessary to avoid underflow and we save this extra step for future work.

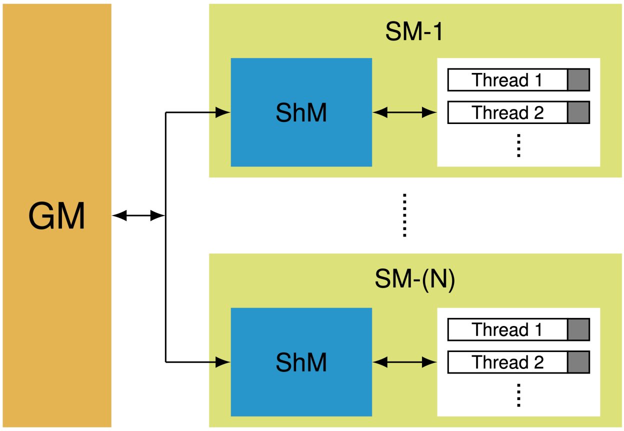

Fig. S1: GPU memory hierarchy. Each GPU is equipped with hundreds to thousands of parallel processors known as streaming multiprocessors (SMs) shown in green. Each SM can execute multiple threads in parallel and threads are further grouped into thread-blocks. Each thread has exclusive access to register memory shown in gray that other threads cannot access. All threads within a thread-block can access the same on-chip shared memory (ShM) shown in blue. GPUs are also equipped with global memory (GM) shown in orange which can be accessed by thread-blocks across all SMs.

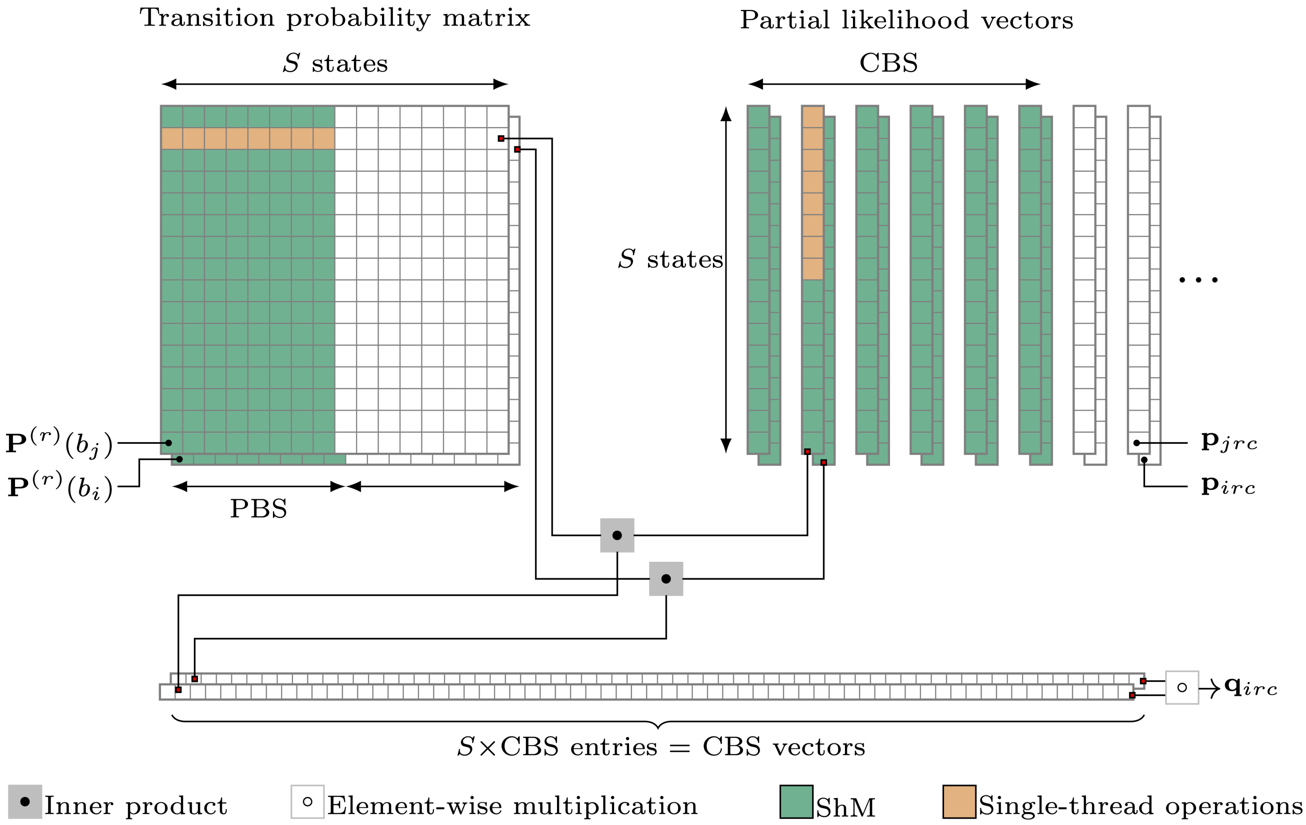

Fig. S2: Parallel thread-block design to compute post-order partial likelihood vectors $\mathbf{p}_{irc}$. One block evaluates column block size (CBS) $\times$ S entries in parallel and prefetches pruning block size (PBS) $\times$ S transition probability entries at time within an inner serial loop.

Table S1. Speed up of parallelizable numerical calculations broken down by individual functions for five MCMC iterations on a GPU compared to a single threaded CPU running on System 3. The percentage of total wall-time consumed by each function on the GPU and CPU is shown in the last two columns. The functions are sorted in descending order of wall-time percentage on the GPU.

State count	Dataset	Function	Speedup	% of wall-time GPU	% of wall-time CPU
4	Carnivores	Copy partials	0.3	39.3	1.3
		Post-order traversal	11.7	19.9	24.0
		Gradient evaluation	15.8	15.8	25.8
		Pre-order traversal	18.7	15.7	30.3
		Other	18.9	9.5	18.5
	Dengue	Copy partials	0.4	38.4	1.4
		Gradient evaluation	14.2	28.8	34.9
		Pre-order traversal	23.7	20.3	41.0
		Post-order traversal	21.6	11.5	21.3
		Other	15.3	1.1	1.4
	Yeast	Post-order traversal	19.6	30.7	46.4
		Copy partials	0.4	27.7	1.0
		Gradient evaluation	17.5	16.9	22.9
		Pre-order traversal	23.6	14.7	26.7
		Other	3.9	10.1	3.1
61	Carnivores	Pre-order traversal	215.8	35.5	40.9
		Gradient evaluation	284.3	28.7	43.5
		Post-order traversal	111.0	23.2	13.8
		Other	49.7	6.8	1.8
		Copy partials	0.2	5.8	0.0
	Dengue	Post-order traversal	91.6	42.5	20.6
		Pre-order traversal	178.7	32.5	30.7
		Gradient evaluation	585.8	13.9	43.1
		Other	178.9	5.9	5.6
		Copy partials	0.4	5.3	0.0
	Yeast	Pre-order traversal	289.8	39.6	38.4
		Gradient evaluation	588.4	27.3	53.9
		Post-order traversal	108.6	20.8	7.6
		Copy partials	0.5	11.4	0.0
		Other	60.9	0.9	0.2

Table S2. Execution time of individual kernels in a single MCMC iteration to infer the branch specific evolutionary rates using a Yang codon model for the carnivores dataset. Time is reported in nanoseconds (ns). 'Matrix transposition' and 'Parallel reduction across nodes' correspond to the 'matrixTranspose' and 'nodeSiteReduction' kernels, respectively.

Name	Number of calls	Time per function call (ns)	Percentage (%)
Pre-order traversal	1,464	177,096	44.62
Gradient evaluation	24	7,515,810	31.04
Post-order traversal	793	126,096	17.21
Matrix transposition	12	126,000	0.26
Parallel reduction across nodes	12	19,027	0.04
Other kernels	846	46,888	6.83

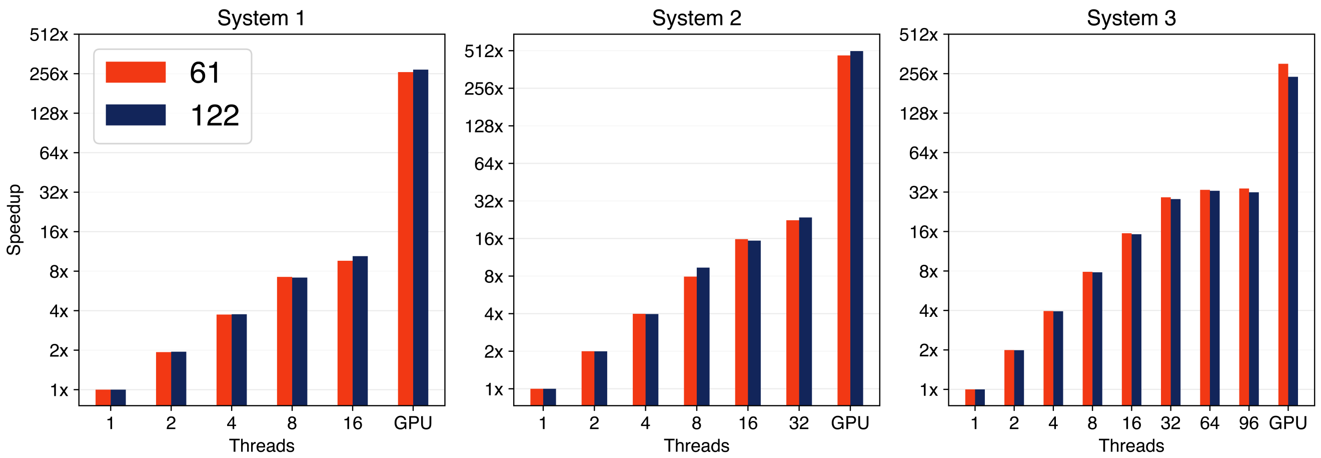

Fig. S3: Speedup of GPU and multi-core CPU instances over a single CPU thread for five MCMC iterations to infer branch-specific evolutionary rates using the yeast dataset for a Yang codon model with a state-space size of 61 and a MMM model with a state-space size of 122. Speedup factors are reported on a log-scale.

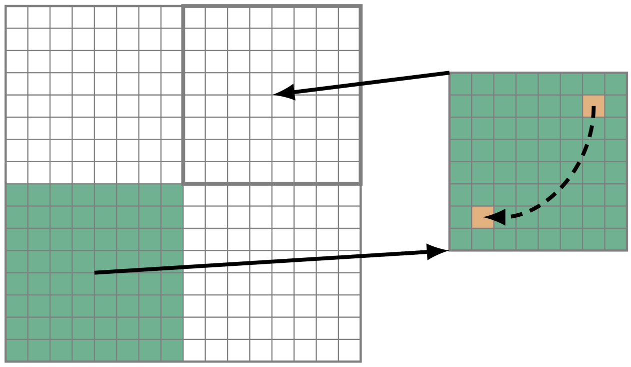

Fig. S4: Parallel thread-block design to calculate the transpose of the finite-time transition probability matrix. One block calculates the transpose of MULTIPLY_BLOCK_SIZE × MULTIPLY_BLOCK_SIZE tile of the matrix.
